# General Eye Remedies

**Published:** 1913-05

**Authors:** W. T. Marrs


For Texas Medical Journal.

### General Eye Remedies.

BY W. T. MARRS, M. D.

The general practitioner has a reluctancy in prescribing for ocular affections in these days when that specialty has reached so high a degree of precision. Yet thousands of people reside in localities where the skilled oculist is inaccessible and the initial treatment, at any rate, must fall upon the general practitioner. That individual should therefore, acquaint himself with the more common inflammatory troubles of the eye, as well as the best method of treating its more common injuries. Iritis and other grave affections, should be early recognized, in order that an oculist may be consulted. Ophthalmia, due to the gonococcus, as well as other forms of micro-organisms, is, as a rule, easily recognized, and should have specific treatment, viz., nitrate of silver and other antiseptic and astringent agencies. Nearly all the other forms of conjunctivitis are quite amenable to treatment, except the phlyctenular variety, which is quite intractable.

Acute inflammatory conditions of the eye demand, above all else, rest or disuse, protection from the light and perfect cleanliness. The best and most specific remedies are futile if the eye is not kept clean, cool and comfortable. Heat is a valuable agency in many forms of acute inflammation and should as a rule be applied in the form of a lavage with the eye cup. Hot water antisepticized with boracic acid is serviceable in many troubles. An inflamed eye should seldom be poulticed, lest the inflammatory process be aggravated. The lavage with hot water alternated with cold, moist applications will often aid in arresting an inflammatory action. Boracic acid and salt solution are very akin to the lachrymal secretion in both chemistry and physiology and a modicum of value may be expected from them in all types of ocular inflammation. The sodium chloride should be used in weak solution in order to avoid irritation.

Cocaine, in a solution of from four to ten per cent strength, is always dependable for the relief of pain whether due to inflammation or injury, but it should not be continued for any great length of time, as it dilates the pupils and also causes a roughening up of the epithelial coat. Weak solutions of morphine and camphor exert a soothing and sedative effect. As an astringent, sulphate of zinc may be employed in a strength of not more than one grain to the ounce. Alum and tannin are safe astringents and may be used with greater freedom than stronger agencies. Lead is a remedy that once had quite a general use, but its value is questionable, and it is even dangerous should there be a crack in the sclera.

Some of these remedies in combination will be highly serviceable in the more common eye affections, but the busy general practitioner cannot always take the time and pains to prepare a solution with exactness and precision. There has been presented to the profession an eye remedy designed to produce palliative and even curative action in the more common affections which the doctor meets almost daily. This remedy is known as Palpebrine and is a well-balanced and accurately compounded prescription. It contains morphine, sulphate of zinc, bichloride of mercury and boracic acid, thus making it a sedative, astringent, alternative and corrective. Palpebrine is not intended as an all-round eye remedy, but it is of value in many ocular affections, giving immediate relief and if persistently used may prove curative in most cases of conjunctivitis and inflammation. The physician who dispenses, and even though he does not dispense, will find this a most valuable remedy to have at his command.

